# Efficacy and safety of rhomboid intercostal block for analgesia in breast surgery and thoracoscopic surgery: a meta-analysis

**DOI:** 10.1186/s12871-022-01599-4

**Published:** 2022-03-16

**Authors:** Ruirong Chen, Sheng Su, Haihua Shu

**Affiliations:** 1 Department of Anesthesiology, Guangdong Provincial People’s Hospital, Guangdong Academy of Medical Sciences, 106 Zhongshan Second Road, Yuexiu District, Guangzhou, Guangdong 510080 P.R. China; 2 The Second School of Clinical Medicine, Southern Medical University, Guangzhou, China

## Abstract

**Background:**

Rhomboid intercostal block (RIB) is a new regional anesthesia technique that provides postoperative analgesia for breast surgery and thoracoscopic surgery. The published papers are not yet fully integrated and do not adequately address the impact and safety of the RIB on postoperative pain.

**Methods:**

The PubMed, Web of Science and Embase were searched from 2016 to 2021 for all available randomized controlled trials (RCTs) that evaluated the analgesic efficacy and safety of RIB after thoracic surgery and breast surgery. Random and fixed-effects meta-analytical models were used where indicated, and between-study heterogeneity was assessed. The primary outcome was Postoperative Numerical Rating Scale (NRS) scores of patients at rest recorded 0–1, 6–8, 24 h after surgery. The secondary outcomes included rate of postoperative nausea and vomiting (PONV), postoperative fentanyl consumption and presence of complications of the block.

**Results:**

From 81 records identified, four studies met our inclusion criteria, including 216 patients (RIB:108 patients; no block: 108 patients). In the primary outcome, RIB group showed significantly lower postoperative NRS at rest at first 0–1 h and 6–8 h (weighted mean difference [WMD] = -1.55; 95% confidence internal [CI] = -2.92 to -0.19; *p* < 0.05), (WMD = -0. 69; 95% CI = -1.29 to -0. 09; *p* < 0. 05). And there was no significant difference between groups in NRS at rest at 24 h (WMD = -0.78; 95% CI = -1.64 to -0.08; *p* = 0.77). Also, RIB group showed significantly lower postoperative NRS of breast surgery and thoracoscopic surgery at 0-1 h (WMD = -3.00; 95% CI = -3.13 to -2.87; *p* < 0.01), (WMD = -1.08; 95% CI = -1.98 to -0.18; *p* < 0.05). In the secondary outcome, the analysis also showed RIB group had significant lower of POVN rates (summary relative risk (RR) = 0.212;95%CI = 0.10 to 0.45; *p* < 0. 01) and the postoperative consumption of fentanyl (WMD = -57.52;95%CI = -106.03 to -9.02; *p* < 0. 05).

**Conclusion:**

This review shows that RIB was more effective in controlling acute pain after breast surgery and thoracoscopic surgery than general analgesia. And it is a trend that RIB may be a kind of effective and safe nerve bock technology and it requires further studies.

## Introduction

Postoperative pain is a significant concern following breast surgery and thoracoscopic surgery [1, 2]. If not treated in time, it may lead to delayed wound healing, respiratory depression, hemodynamic disorders, anxiety and other complications, and ultimately lead to difficult recovery of patients [3–5]. Therefore, the prevention of postoperative pain is of great importance for patients. Regional anesthesia can provide good postoperative pain management and may reduce the incidence of chronic pain [6]. The Rhomboid intercostal block (RIB) was first proposed by Elsharkawy et al. in 2016 [7].The block provides analgesia for the anterior and posterior thorax following the injection of a local anesthetic in the fascial plane between the rhomboid major and the intercostal muscles. There have been some case reports and cohort studies describing its use. Still, the effectiveness of the new technique is controversial. In recent years, more and more randomized controlled trials have been published. Published articles have not been well integrated, and the effects of RIB on postoperative pain and its safety have not been fully described. Therefore, we conducted a systematic review and meta-analysis to evaluate the efficacy and safety of RIB.

## Methods

This meta-analysis was performed according to the Preferred Reporting Items for Systematic Reviews and Meta-Analyses (PRISMA) statement [8]. We searched for randomized trials that compared the efficacy and safety of rhomboid intercostal block for analgesia in breast surgery and thoracoscopic surgery.

### Inclusion and exclusion criteria

The inclusion criteria of the study were listed as follows: the study design was RCT, the subjects were patients undergoing thoracoscopic surgery or breast surgery, the comparison was between RIB and the no block group. The exclusion criteria of the study were listed as follows: the types of articles were review, case report, experiment of animal, comments, letter and vitro studies, the type of the surgery was not thoracoscopic surgery or breast surgery, RIB was not mentioned. In the case of repeated studies by the same authors, the most recent or most informative was included. Abstracts were not considered unless the full-text studies were available, and only English and English-translated studies were used.

### Search strategy

We searched PubMed, Web of Science databases and Embase to identify RIB relevant articles. A reference list of references that might qualify was also manually searched to identify additional trials that met the inclusion criteria. Trials published from 2016 until 2021 were included in the analysis. The search terms included the following: rhomboid intercostal block, thoracoscopic surgery and breast surgery. Appropriate adjustments were made when searching the database.

### Selection of included studies

Two authors (Ruirong Chen and Sheng Su) independently screened the titles and abstracts of all identified articles. When necessary, reviewed the full report to identify potential relevant research. Any difference between the two reviewers was discussed until a consensus was reached. If two independent reviewers cannot reach an agreement, a third reviewer (Haihua Shu) made the final decision.

### Data extraction

A data extraction form was created using a Microsoft Excel spreadsheet. The data was then extracted independently by two reviewers (Ruirong Chen and Sheng Su). The reviewers discuss any differences in data extraction until an agreement is reached. If two independent examiners cannot agree, a third reviewer (Haihua Shu) made the final decision.

The data extraction form collected information regarding the following data from each study: first author, year of publication, study population characteristics, study design, inclusion and exclusion criteria, type of operation, number of patients enrolled in each type of surgery, intervention performed, and the reported outcome.

### Assessment of risk of bias

Two authors independently assessed risk of bias at the study level using the Cochrane risk-of-bias tool [9]. The quality of evidence for each outcome was assessed using the GRADEpro guideline development tool [10]. The reviewers divided the strength of evidence into high quality (⊕ ⊕  ⊕ ⊕) medium quality (⊕ ⊕  ⊖ ⊖) low quality (⊕ ⊖  ⊖ ⊖) or very low quality (⊖ ⊖  ⊖ ⊖) evidence. All quality assessments are conducted in duplicate by two independent reviewers (Ruirong Chen and Sheng Su) to discuss any differences in quality assessments until consensus is reached. If two independent reviewers cannot reach consensus, the final decision is made by a third reviewer (Haihua Shu).

### Primary and secondary outcome

The primary outcome was Postoperative Numerical Rating Scale (NRS) scores of patients at rest recorded 0–1, 6–8, 24 h after surgery. The secondary outcomes included rate of postoperative nausea and vomiting (PONV), postoperative fentanyl consumption and presence of complications of the block.

### Data processing

The morphine and sufentanil dose were converted to the equivalent dose of fentanyl by clinically derived mean relative potency dose ratio to achieve the standardization of the analytical dose [11]. For incomplete data, the reviewers attempted to contact the authors of the original articles by email to request for further and complete data. If data were expressed in terms of median and interquartile range, conversion to mean and standard deviation was done using Hozo’s validated formula [12]. Since the NRS scores is consistent with the visual analogue scale (VAS) scores, the values of the VAS scores are converted to the NRS scores values for comparison [13, 14].

### Statistical analysis

The statistical analysis of the pooled data was performed using RevMan5.2 and Stata 19.0. For binary variables, relative risk (RR) and 95% confidence interval (CI) was used. For continuous variables, weighted mean difference (WMD) and 95% confidence interval (CI) were used. Heterogeneity was quantified using the Cochrane I [2] statistics. I [2] is expressed as a percentage value; the higher the proportion, the higher the degree of heterogeneity. The random-effects model was used if there was heterogeneity between studies; otherwise, the fixed-effects model was used. For all outcomes, the statistical significance was set to *P* < 0.05 and with 95% confidence intervals (CI).

### Assessment of publication bias

Because of the small number of eligible trials, we did not assess publication bias.

## Results

**Fig. 1:**
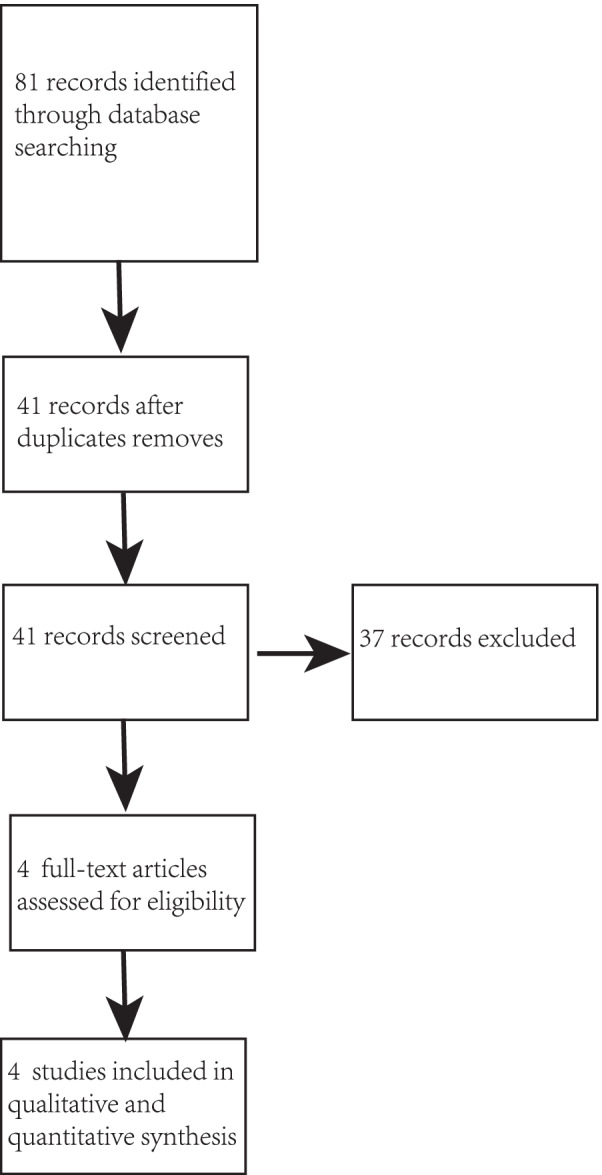
Flow diagram of included and excluded studies

We identified 81 records through database searching and screened their summaries for eligibility, after removing the duplicates, there were still 41 unique articles remaining. Of 41 full-text articles assessed, we identified four separate studies. We used data from several secondary analyses of these studies in this meta-analysis. A total of 216 patients were enrolled in 4 included studies [15–18], 108 in the RIB group, 108 in the control group with no blocks. The strategy of the research and the process of the selection were shown in the flow diagram of Fig. 1.

All 4 [15–18]studies were randomised controlled trials published in peer reviewed journals. Two trials [17, 18] compared RIB with no block. One trials [15] compared RIB both to no block and to RIB combined with sub‑serratus plane block, and one trials [16] compared RIB both to no block and to Type-II pectoral nerve block. Their results were analyzed separately. The detailed features of the included studies (4 RCTs) are listed in Table 1.

**Table 1:** Characteristics of included studies

	**Group R**				**Group C**			
study	Başak Altıparmak 2020	Wei Deng 2021	Bahadir Ciftci 2021	Amarjeet Kumar 2020	Başak Altıparmak 2020	Wei Deng 2021	Bahadir Ciftci 2021	Amarjeet Kumar 2020
number of patients	28	30	30	20	28	30	30	20
Age (years)	53.8 ± 11.2	60.5 ± 11.6	50 ± 3.75	9.6 ± 1.6	52 ± 11.5	55.6 ± 11.5	42 ± 4.25	10.26 ± 1.28
Weight (kg)	72.6 ± 8.0	-	70 ± 2.5	35.43 ± 5.17	70.4 ± 9.9	-	70 ± 3	34.16 ± 3.7
Hight (cm)	160.2 ± 5.1	-	161 ± 2.5	-	160.4 ± 5	-	163 ± 2.25	-
Body mass index (kg/m^2^)	28 ± 2.80	23.2 ± 2.9	-	-	27 ± 3.4	24.3 ± 2.8	-	-
American Society of Anesthesiologists (I/II)	22/5	13/17	16/14	-	22/5	12 /18	15/15	-
Duration of anesthesia (min)	-	125.1 ± 38	100 ± 5	-	-	116.5 ± 25.6	95 ± 6.25	-
Duration of Operation (min)	104.2 ± 9.2	139.7 ± 35.3	80 ± 3.75	89.5 ± 21.61	106.3 ± 9.6	141.4 ± 31.2	77.5 ± 5	93.33 ± 15,50
Surgery type	breast cancer surgery	thoracoscopic surgery	breast cancer surgery	thoracoscopic surgery	breast cancer surgery	thoracoscopic surgery	breast cancer surgery	thoracoscopic surgery

**Fig. 2:**
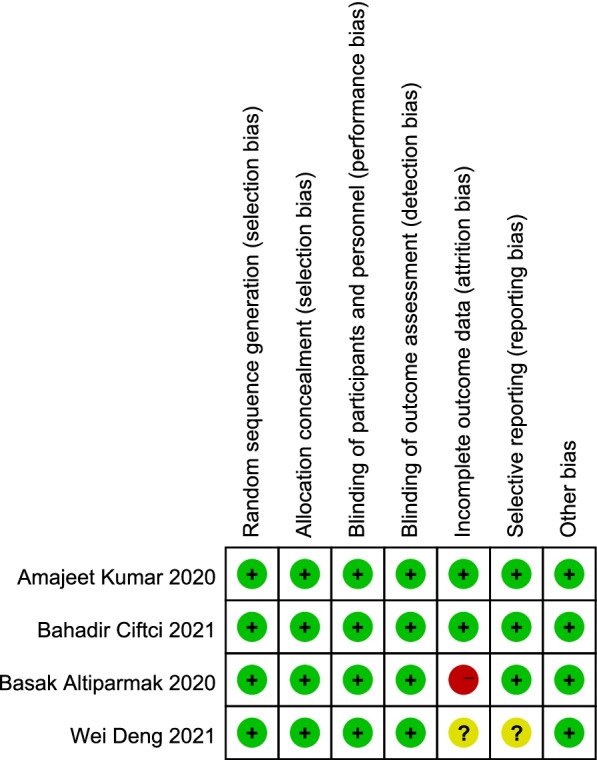
Risk of bias of randomised controlled trials. Green circle, low risk; yellow circle, some concerns; red circle, high risk

The results of the risk-of-bias assessment are summarised in Fig. 2. As per the inclusion criteria, all the studies included in the meta-analysis are randomised controlled trials.

### Primary outcome

For postoperative pain scores, three studies used NRS scores [15, 17, 18] and one used VAS scores [16]. Since the NRS score was consistent with the VAS score, the VAS score value was converted to the NRS score value for comparison [13, 14].

**Fig. 3:**
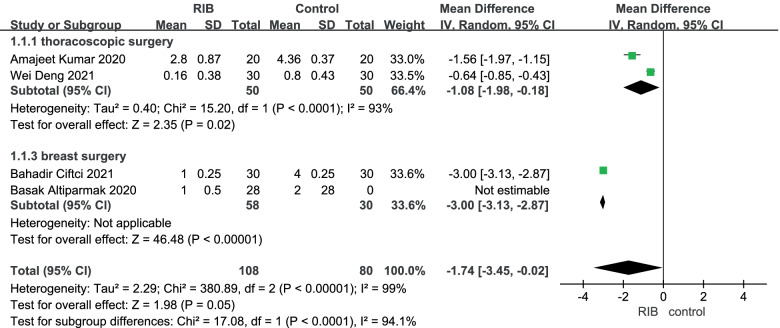
Forest plot of comparison: rhomboid intercostal block (RIB) vs. no block, postoperative Numerical Rating Scale (NRS) scores at rest at the first 0–1 h. IV, inverse variance

NRS at rest at the first 0–1 h: Four trials [15–18] reported the NRS at rest at the first 0–1 h and the random-effect model was used to analysis the outcome of them. The results showed that compared to no block group, RIB resulted in significantly lower NRS at rest at the first 0–1 h (WMD = -1.55; 95% CI = -2.92 to -0.19; *p* < 0.05) with significant heterogeneity among the studies (I^2^ = 99%, heterogeneity *p* < 0.0001) (Fig. 3).

Sensitivity analysis found that significant heterogeneity remained between trials after alternating omissions of a study. Then we performed the subgroup analysis to investigate that whether the NRS at rest at the first 0–1 h was influenced by the subjects of different surgeries. Of the 4 trials [15–18], 2 [15, 17] were about thoracoscopic surgery, which were set as subgroup 1. Compared with no block, RIB showed a significant lower NRS at resting time of 0–1 h (WMD = -1.08; 95% CI = -1.98 to -0.18; *p* < 0.05) and there was significant heterogeneity among the studies (I^2^ = 93%, heterogeneity *p* < 0.0001). Meanwhile, two other studies [16, 18] performed breast surgery and were set as subgroup 2. It showed a significant reduction in NRS at rest in RIB group during the initial 0–1 h resting period (WMD = -3.00; 95% CI = -3.13 to -2.87; *p* < 0.0001). The heterogeneity was no applicable (Fig. 3).

**Fig. 4:**
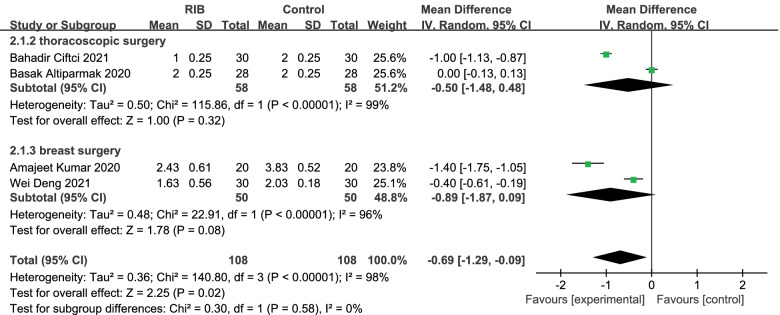
Forest plot of comparison: rhomboid intercostal block (RIB) vs. no block, postoperative Numerical Rating Scale (NRS) scores at rest at the first 6–8 h. IV, inverse variance

NRS at rest at the first 6 h to 8 h: The results showed that compared to no block group, RIB resulted in significantly lower in NRS at rest at the first 6 h to 8 h (WMD = -0. 69; 95% CI = -1.29 to -0. 09; *p* < 0. 05) with significant heterogeneity among the studies (I^2^ = 98%, heterogeneity *p* < 0. 0001) (Fig. 4).

Our sensitivity analysis found that significant heterogeneity remained between trials after alternating omissions of a study. Then we perform the subgroup analysis to investigate that whether the NRS at rest at the first 6–8 h was influenced by the subjects of different surgeries. Of the 4 trials 15–18], 2 studied [15, 17] thoracoscopic surgery, which were subgroup 1. Compared to no block group, RIB showed a lower NRS at rest time of first 6–8 h, but no significant (WMD = -0.50; 95% CI = -1.48 to 0.48; *p* = 0.32) and there was significant heterogeneity among the studies (I^2^ = 99%, heterogeneity *p* < 0.0001). Meanwhile, two other studies [16, 18] performed breast surgery and were classified as subgroup 2. It also showed a reduction in NRS at rest in those who received RIB during the 6–8 h resting period but no significant (WMD = -0.89; 95% CI = -1.87 to 0.09; *p* = 0.08) with significant heterogeneity among the studies (I^2^ = 96%, heterogeneity *p* < 0. 0001) (Fig. 4).

**Fig. 5:**
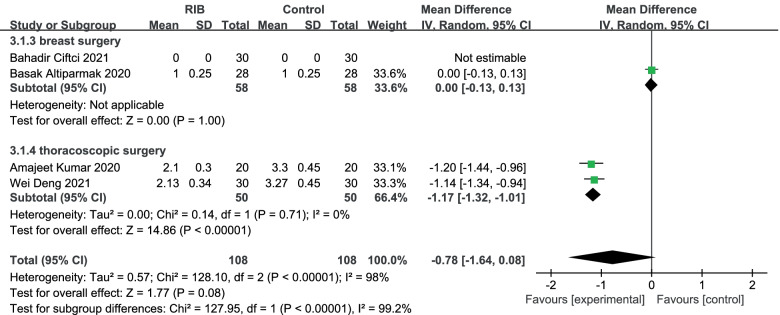
Forest plot of comparison: rhomboid intercostal block (RIB) vs. no block, postoperative Numerical Rating Scale (NRS) scores at rest at the first 24 h. IV, inverse variance

NRS at rest at the first 24 h: We used the random-effect model to analysis the outcome of NRS at rest at the first 24 h in four trials [15–18]. The results showed that compared to no block group, RIB resulted in no significantly difference in NRS at rest at 24 h (WMD = -0.78; 95% CI = -1.64 to 0.08; *p* = 0.77) with significant heterogeneity among the studies (I^2^ = 98%, heterogeneity *p* < 0. 0001) (Fig. 5).

Our sensitivity analysis found that significant heterogeneity remained between trials after alternating omissions of a study. Then we perform the subgroup analysis to investigate that whether the NRS at rest at the first 24 h was influenced by the subjects of different surgeries. Of the 4 trials [15–18], 2 studied [15, 17] thoracoscopic surgery, which were subgroup 1. There was no significant difference between groups in NRS at resting time of first 24 h (WMD = 0; 95% CI = -0.13 to 0.13; *p* = 1.00). The heterogeneity was no applicable. Meanwhile, two other studies [16, 18] performed breast surgery and were classified as subgroup 2. It also showed significant reduction in NRS at rest in those who received RIB at resting time of first 24 h (WMD = -1.17; 95% CI = -1.32 to -1.01; *p* < 0. 0001) with no heterogeneity among the studies (I^2^ = 0%, heterogeneity *p* = 0.71) (Fig. 5).

### Secondary outcome

**Fig. 6:**
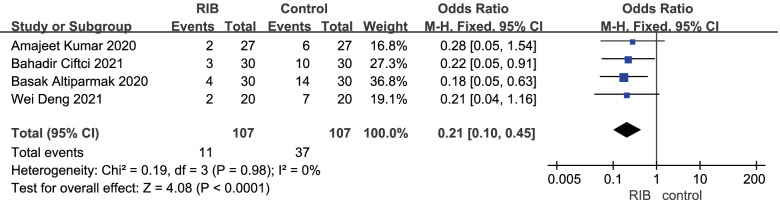
Forest plot of comparison: rhomboid intercostal block (RIB) vs. no block, postoperative nausea and vomiting (PONV). IV, inverse variance

Meta-analysis of rate of PONV: PONV data came from four trials [15–18]. The results of our analysis are shown in Fig. 6. In conclusion, the incidence of PONV in RIB and no block groups was 10.30% and 34.60%, respectively. There was significant difference in PONV between RIB and no block group (OR = 0.212;95%CI = 0.100 to 0.447; *p* < 0. 0001), no evidence of high heterogeneity was found between the included studies (I^2^ = 0%; *p* < 0. 0001).

**Fig. 7:**
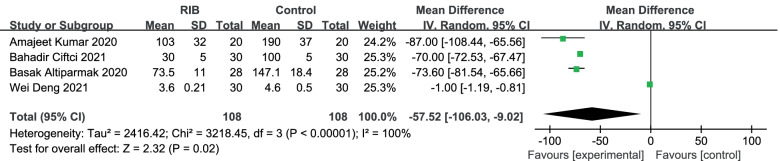
Forest plot of comparison: rhomboid intercostal block (RIB) vs. no block, postoperative fentanyl use. IV, inverse variance

Meta-analysis of rate of postoperative fentanyl consumption: In terms of postoperative analgesic use, two studies used fentanyl, one morphine [17], and one sufentanil [15]. So, we converted morphine and sufentanil doses from the other two studies to fentanyl doses. We used a random-effects model to analyze the results of postoperative fentanyl use. Results in four trials [15–18] showed that RIB resulted in a significant reduction in postoperative fentanyl compared to the no block group (WMD = -57.52;95%CI = -106.03 to -9.02; *p* < 0. 05), there was heterogeneity in the study (I^2^ = 100%, heterogeneity *p* < 0. 00,001) (Fig. 7).

**Fig. 8:**
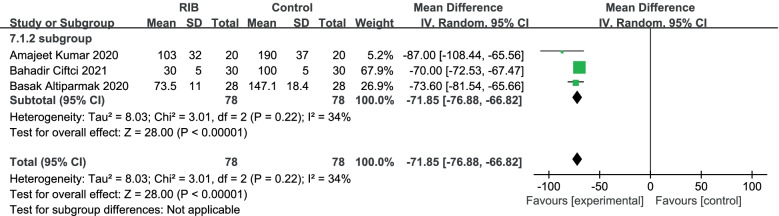
Forest plot of comparison: rhomboid intercostal block (RIB) vs. no block, postoperative fentanyl use, excluded a trial with minimum sufentanil. IV, inverse variance

And we excluded a trial that used sufentanil as an indicator because the dose of sufentanil converted to fentanyl was much lower than in the other groups. Results in three trials [16–18] showed that RIB resulted in a significant reduction in postoperative fentanyl compared to the no block group (WMD = -71.85;95%CI = -76.88 to -66.82; *p* < 0. 00,001), there was no heterogeneity in the study (I2 = 34%, heterogeneity *p* = 0.22) (Fig. 8).

The rate of presence of complications of the block: four trials [15–18] showed that none of the patients had block-related complications in group.

## Discussion

This meta-analysis of 4 RCTs included 216 patients to evaluate the analgesic efficacy and safety of RIB in thoracoscopic surgery and breast surgery. RIB was more effective in controlling acute pain after breast surgery and thoracoscopic surgery than intravenous analgesia. Preoperative RIB significantly reduced pain scores at early time points and reduced 24-h opioid consumption. The reduced difference in pain scores between the two groups at the later time point (24 h postoperatively) may be result of an increase in opioid intake.

RIB is a new interfascial plane block which can provide analgesia between the T2 and T9 dermatomes [7]. Earlier cadaveric studies suggested that the spread of the dye in a cadaver which revealed extensive craniocaudal and anteroposterior spread, potentially accounting for the effectiveness of the block [7, 19]. As previously reported, RIB has been shown to be effective in both thoracoscopic and breast surgery [20–25].

The results of our review are limited by considerable heterogeneity. Our sensitivity analysis found that significant heterogeneity remained between trials after alternating omissions of a study. Then we performed the subgroup analysis, but they still existed the heterogeneity. And Our subgroup analysis showed that early RIB had a favorable effect in both types of surgery. Data of the time to first postoperative analgesic request was given in only one trial [15]: the time to first postoperative analgesic request in the RIB group was significantly longer than that in control group (*p* < 0.001).

In terms of NRS, the RIB group showed significant lower scores than the no block group in 0–1 and 6–8 h, which mean lower pain level in RIB group. Although there was no significant difference between groups in NRS at resting time of first 24 h, RIB showed excellent postoperative analgesic effect, which may be beneficial to early postoperative rehabilitation of patients.

In terms of postoperative complications, several studies have shown that the RIB group has fewer postoperative complications. The most common complication was PONV, and there was a significant difference between the RIB and no block groups. The incidence of PONV was 10.30% in the RIB group and 34.60% in the no block group, respectively. This may be due to low fentanyl consumption in the RIB group. Nausea and vomiting is mainly result of vagus nerve excitation, hypotension, distension of the stomach and the use of opioids. RIB does not affect the vagus nerve, has little effect on hemodynamics, and the use of opioids after surgery is relatively rare, so the incidence of vomiting is relatively low.

As known, Opioid tolerance (increased dose needed for analgesia) and opioid-induced hyperalgesia (OIH) (paradoxical increase in pain with opioid administration) can contribute to both poorly controlled pain and dose escalation. RIB could reduce fentanyl consumption, which may do help to prevent OIH [26, 27]. In addition, low fentanyl consumption in the RIB group may bring other potential benefits, such as low risk of constipation, itching and others [28]. In short, low fentanyl consumption may contribute to enhanced recovery after surgery.

Postoperative respiratory depression can be caused by pain. In addition to discouraging the sufferer from inhaling deeply, reducing tidal volume and increasing respiratory rate, it may also suppress the cough reflex. Postoperative analgesia is an effective measure to prevent respiratory depression, atelectasis and pulmonary infection [29, 30]. One of the studies reported the occurrence of respiratory depression in the RIB group and the no block group. The rate of respiratory depression was 10% in the RIB group and 30% in the no block group, which showed a significant difference. Other complications were not reported in the included studies. Block-related complications did not occur in any of the studies. Therefore, we have to conclude that RIB is a relatively safe blocking technique.

The erector spinae plane block (ESP) is a relatively new technique that was first described by Forero et al. in 2016 [31]. And it's proven to be effective in breast surgery and thoracoscopic surgery [32, 33]. The injection site of RIB is more peripheral than that used with ESP, and the spread of local anaesthetic runs mostly towards the lateral branches of the intercostal nerves rather than to the paravertebral and epidural space. Because the sympathetic chain blockade is not as deep with RIB as compared with ESP, the incidence of hypotension could reduce. Since there is no study about comparing ESP and RIB, we suggest future studies to determine if RIB is non-inferior to ESP.

Future studies should also incorporate better double- blinding techniques and sham controls which were lacking in the current studies. There is also no study comparing paravertebral block and RIB. Given the higher risk profile of paravertebral blocks [34], we also suggest future studies to determine if RIB is non-inferior to paravertebral block.

There are several limitations to this study. First, although sensitivity analysis and subgroup analysis were used to reduce heterogeneity, there was still significant heterogeneity in some subgroups. One of the 4 studies included children (7–12 years) which are differently from the adult patients. In pediatric age the sensitive to pain is different from adults [35, 36]. Thus, there was heterogeneity between children's scores and adults' scores on the pain scale. But compared with the control group, postoperative pain scores and fentanyl use were significantly reduced in the RIB group both adults and children. Second, many of the included studies only provided data on PONV, but not on other common complications (bleeding, arrhythmias, postoperative respiratory system, etc.). Only one study compared respiratory depression between RIB and the no block group which may related to the type of surgery varies from study to study. Therefore, we were unable to make a comprehensive assessment of postoperative safety between them. Third, pain is known as a complex physiological and psychological activity, and mechanism of analgesia therefore may not be entirely linked to the provision of a conduction nerve block. Therefore, sensory exams are important before nerve block. However, all included studies lacked. In addition, two study excluded patients with history of chronic pain requiring analgesics and another two did not specify whether excluded patients with chronic pain. This may make a subtle difference to the final result. Finally, compared with other meta-analyses, the sample size of the studies we included was small, which may weaken our conclusions. Large sample and multicenter RCTs should be performed for further discussion.

## Data Availability

The datasets used and/or analyzed during the current study are available from. the corresponding author on reasonable request.
