# Correction to: Efficacy and safety of rhomboid intercostal block for analgesia in breast surgery and thoracoscopic surgery: a meta-analysis

**DOI:** 10.1186/s12871-022-01643-3

**Published:** 2022-04-08

**Authors:** Ruirong Chen, Sheng Su, Haihua Shu

**Affiliations:** 1 Department of Anesthesiology, Guangdong Provincial People’s Hospital, Guangdong Academy of Medical Sciences, 106 Zhongshan Second Road, Yuexiu District, Guangzhou, Guangdong 510080 P.R. China; 2 The Second School of Clinical Medicine, Southern Medical University, Guangzhou, China

**Correction to: BMC Anesthesiol 22, 71 (2022)**

**https://doi.org/10.1186/s12871-022-01599-4**

Following publication of the original article [1], the authors reported an error in the affiliation 2.

The incorrect affiliation is: The Second School of Clinical Medicine, Southern Medical University, 106 Zhongshan Second Road, Yuexiu District, Guangzhou, Guangdong 510080, P.R. China.

The correct affiliation is: The Second School of Clinical Medicine, Southern Medical University, Guangzhou, China.

The original article [1] has been updated.

## References

[1] Chen R, Su S, Shu H (2022). Efficacy and safety of rhomboid intercostal block for analgesia in breast surgery and thoracoscopic surgery: a meta-analysis. BMC Anesthesiol.

